# Prognosis in meningoencephalitis of unknown origin in dogs: Risk factors associated with survival, clinical relapse, and long‐term disability

**DOI:** 10.1111/jvim.17037

**Published:** 2024-03-14

**Authors:** Rita Gonçalves, Steven De Decker, Gemma Walmsley, Thomas W. Maddox

**Affiliations:** ^1^ Department of Veterinary Science Small Animal Teaching Hospital, University of Liverpool Neston UK; ^2^ Department of Musculoskeletal and Ageing Science Institute of Lifecourse and Medical Sciences, University of Liverpool Neston UK; ^3^ Department of Clinical Science and Services Royal Veterinary College, University of London Hatfield UK

**Keywords:** canine, mortality, MUO, outcome, prognosis, relapse

## Abstract

**Background:**

Meningoencephalitis of unknown origin (MUO) comprises a group of noninfectious inflammatory diseases affecting the central nervous system of dogs. Previous studies have reported individual risk factors for survival but prognostication for MUO remains challenging.

**Objectives:**

Identify clinical prognostic variables in dogs with MUO.

**Animals:**

A retrospective study of 447 dogs presented to 2 UK referral hospitals and diagnosed with MUO.

**Methods:**

Medical records of dogs diagnosed with MUO were retrospectively reviewed. Multivariable logistic regression was used for the identification of risk factors for survival and Cox proportional hazards analysis for the identification of risk factors for clinical relapse.

**Results:**

Eighty‐two percent (366/447) of dogs with presumptive MUO survived to discharge and 63.5% (284/447) were alive at 6 months; 36% of the latter (103/284) had persistent neurological deficits. Breed (pugs; *P* = .03), epileptic seizures (*P* < .001), paresis (*P* < .001), and higher neurodisability scale (NDS) score (*P* < .001) at presentation were negatively associated with survival to 6 months. Dogs with persistent deficits had higher NDS scores on presentation (*P* = .001). Median follow‐up time was 11 months (interquartile range [IQR], 1‐24) and 50.6% (160/316) relapsed during treatment (median time to relapse, 7 months; IQR, 2‐15). Incomplete resolution of the clinical signs during the 6 months after diagnosis (*P* < .001), higher NDS score (*P* < .001), and longer duration of the clinical signs (*P* < .001) were associated with relapse.

**Conclusions and Clinical Importance:**

Knowledge of risk factors associated with survival, incomplete recovery and clinical relapse in MUO can help guide monitoring and treatment and improve owner communications regarding prognosis for this debilitating disease.

**Abbreviations**
AUC: area under the curve
CI: confidence intervals
CSF: cerebrospinal fluid
GFAP: glial fibrillary acidic protein
ICP: intracranial pressure
IQR: interquartile range
MRI: magnetic resonance imaging
MS: multiple sclerosis
MUO: meningoencephalitis of unknown origin
NDS: neurodisability scale
NME: necrotizing meningoencephalomyelitis
ROC: receiver operating characteristic
TNCC: total nucleated cell count

## 1 INTRODUCTION

Meningoencephalitis of unknown origin (MUO) in dogs is a heterogeneous disorder with a broad spectrum of clinical signs and presentations. It comprises a group of idiopathic inflammatory diseases that can only be definitively diagnosed by histopathologic evaluation of the brain, including granulomatous meningoencephalomyelitis, necrotizing meningoencephalomyelitis, and necrotizing leukoencephalitis.^1^, ^2^ The true incidence of these conditions remains uncertain but, in referral populations, MUO seems to be the most common inflammatory condition affecting the central nervous system in dogs.^3^ The wide range of clinical signs, imaging findings, and pathologic lesions in affected dogs makes studying MUO in a clinical population challenging, and reported outcomes therefore remain variable.

Despite initiation of appropriate treatment, 25% to 33% of dogs with MUO are reported to die within 1 week of diagnosis,^4^, ^5^ and 31% within 100 days of diagnosis.^6^ Previous studies have identified that younger age at diagnosis^7^ and early diagnosis (within 7 days of development of clinical signs)^8^ are associated with longer survival times, whereas seizures^5^, ^9^, ^10^ and obtundation^5^, ^6^, ^10^ are associated with shorter survival times. Recently, a neurodisability scale (NDS) has been shown to be a reliable clinical assessment tool for dogs with MUO, but initial data from a small population of dogs did not show an association between their scores and outcome.^11^

Although some information regarding the prognosis for survival in MUO is available, most studies include relatively small numbers of dogs and findings have not been consistent across studies. Treatment efficacy may be improved by identifying specific MUO subpopulations at higher risk of treatment failure or relapse despite having received disease‐modifying treatments. We, therefore, aimed to evaluate a large population of dogs with MUO to identify clinical variables associated with prognosis: survival at 6 months after diagnosis, persistent neurodisability, and clinical relapse.

## 2 MATERIALS AND METHODS

The medical records of dogs diagnosed with MUO at the Small Animal Teaching Hospital (SATH) of the University of Liverpool and the Royal Veterinary College (RVC) were retrospectively reviewed. Ethical approval for use of data was granted by the Ethics Committee of the University of Liverpool (VREC840). Dogs meeting the following criteria were included in the study: (1) clinical examination consistent with focal or multifocal intracranial neuroanatomical localization; (2) >6 months of age; (3a) multiple, single or diffuse intra‐axial hyperintensities on T2‐weighted magnetic resonance imaging (MRI) and mononuclear pleocytosis (total nucleated cell count [TNCC] >5/μL) on cerebrospinal fluid (CSF) analysis, or (3b) normal brain MRI but mononuclear pleocytosis. In dogs with MRI findings consistent with MUO (3a) but with signs of increased intracranial pressure (ICP) on imaging (namely transtentorial or foramen magnum herniation), CSF collection was considered contraindicated and not performed, but these cases were included. Dogs were excluded if the TNCC was normal on CSF analysis (independent of their MRI findings), if follow‐up time was <6 months (except for those dogs dying during this period), or if the owners elected to euthanize at the time of diagnosis without treatment. The MRI examinations were performed using a 1.5 T (Philips Ingenia CX, Philips Healthcare) or a 1 T (Siemens Magnetom, Siemens Healthcare) scanner.

The following data were extracted from the medical records: age, sex, neuter status, breed, year of presentation, season of the year, onset, duration and progression of the clinical signs before diagnosis, neurological deficits identified on presentation, neurolocalization (categorized as forebrain, brainstem, cerebellum, central vestibular [used when clinical signs were insufficient to localize as brainstem or cerebellum], optic nerves/chiasm, multifocal, or no neurological deficits when only pain was identified), and survival to discharge. The findings of the neurological examination recorded in the patient files were used to retrospectively assign the NDS^11^ for each dog (calculated by the same assessor in all dogs). Onset of clinical signs was categorized as acute (<24 hours), subacute (between 24 and 72 hours), and chronic (>72 hours) and duration of the clinical signs was ≤7 days or >7 days before presentation. The results of the CSF analysis (TNCC and protein concentration) also were recorded, as well as the treatment received. The initial treatment received also was recorded and further categorized as corticosteroids alone, corticosteroids and IV cytosine arabinoside (200 mg/m^2^) or corticosteroids and SC cytosine arabinoside (200 mg/m^2^). Long‐term treatment was categorized as corticosteroids alone, corticosteroids and repeated administrations of SC cytosine arabinoside for a minimum of 4 treatment cycles, and corticosteroids and another PO immunomodulatory drug. For statistical analysis, breeds with >15 individuals represented were compared with the remainder of the study population.

The outcome was assessed at the last follow‐up appointment or telephone conversation with the owners or referring veterinary surgeons. Episodes of suspected relapse (recurrence of similar or new neurological deficits and, when possible, repeat MRI of the brain and CSF analysis) were recorded as well as response to rescue treatment when available.

Statistical analysis was performed using the software SPSS 27.0 (SPSS Inc., Chicago, Illinois). Continuous data were assessed for normality using the Shapiro‐Wilk test. Descriptive statistics are reported for continuous variables using mean (SD) for approximately normally distributed variables and median (interquartile range [IQR]) for variables with skewed distributions, and frequencies (with 95% confidence intervals [CI] where appropriate) are reported for categorical variables. The Mann‐Whitney test was used to assess the association between the NDS score and resolution of clinical signs.

Univariable binary logistic regression was performed to identify clinical variables associated with survival at 6 months after diagnosis (using the information from last examination or telephone conversation). Before multivariable analysis, all variables were assessed for correlation using Spearman's rank correlation coefficients. If Spearman's rank correlation coefficient was >.8, the most statistically significant or biologically plausible variable was selected. Any independent variable demonstrating some association on preliminary univariable analysis (*P* < .2) was considered for inclusion in a multivariable model. Multivariable models were constructed using a manual backwards stepwise removal approach; variables with *P* < .05 were retained, with model fit assessed using the Hosmer‐Lemeshow statistic. Cox proportional hazards analysis was used to identify clinical variables associated with long‐term relapse using the same approach, with the assumption of proportional hazards assessed by evaluating the significance of incorporating time‐dependent covariate interaction terms and evidence for improved model fit, and by inspection of the Schoenfeld residuals of the final model. The receiver operating characteristic (ROC) curve was used to determine the usefulness of the NDS score for predicting survival at 6 months after diagnosis of MUO; the corresponding area under the curve (AUC) was calculated to evaluate the predictive capability and the Youden index was used to calculate the optimal cut‐off value. Survival times were assessed using Kaplan‐Meier plots and were compared between groups by log‐rank analysis.

## 3 RESULTS

**FIGURE 1:**
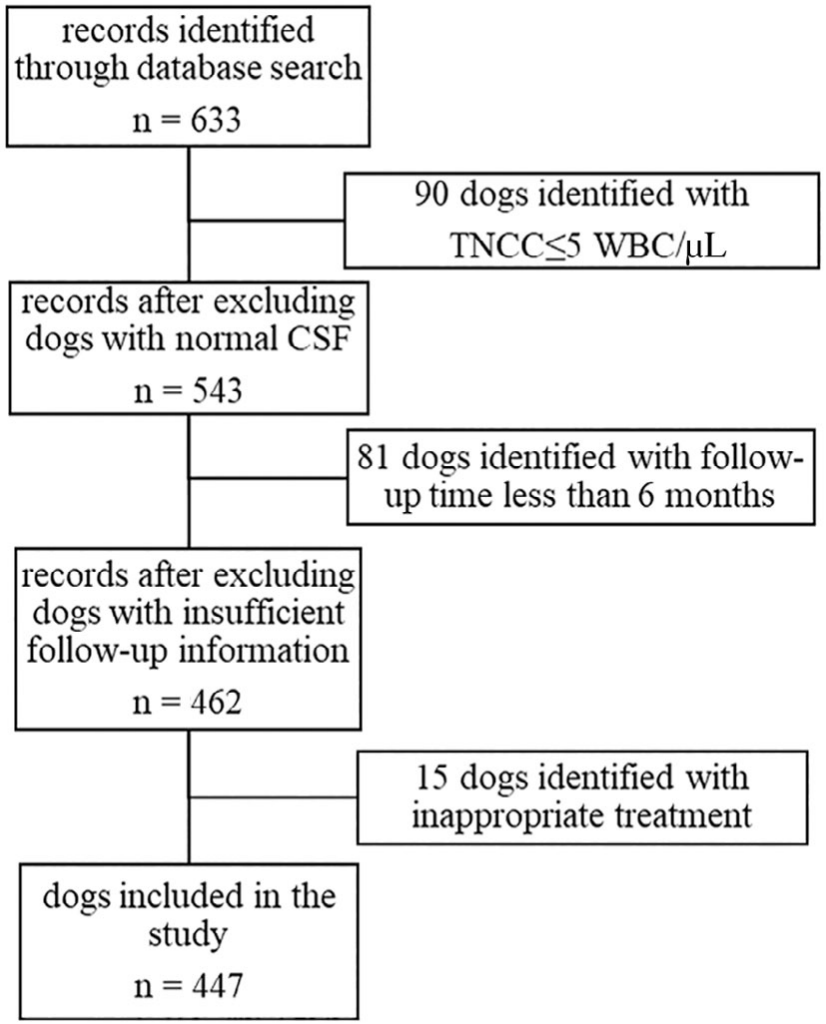
Study protocol for data analysis. n, number of dogs.

A total of 633 client‐owned dogs diagnosed with suspected MUO were initially identified from the hospital records of the 2 contributing institutions over a 12‐year period (2010‐2022). After a review of the patient files, 447 dogs met the inclusion criteria (Figure 1).

The median age at presentation was 48 months (IQR, 26‐73). There were 246 (55%) females (145 neutered) and 201 (45%) males (116 neutered). The most frequently affected breeds after crossbreeds (n = 64) were Chihuahua (n = 55), French bulldog (n = 40), pug (n = 33), Maltese terrier (n = 33), West Highland white terrier (n = 29), Yorkshire terrier (n = 20), Boston terrier (n = 17), Labrador retriever (n = 16), Jack Russell terrier (n = 14), Shih Tzu (n = 13), Lhasa Apso, and English springer spaniel (n = 9), Pomeranian and Poodle (n = 8), Bichon Frise and Cavalier King Charles spaniel (n = 7) and 45 breeds were presented with <5 dogs. The cases presented evenly across the 12 years of study (between 5.8% and 10.7% each year) and throughout the seasons, with 24.4% presenting in winter, 24.2% in spring, 26.2% in summer, and 24.9% in autumn. The demographic and clinical characteristics of the study population are summarized in Table 1 and the findings of the neurological examination in Table 2. Necropsy confirmation was available in 24 cases (17 granulomatous meningoencephalitis and 7 necrotizing meningoencephalitis).

**TABLE 1:** Descriptive statistics for clinical and demographic information for the 447 dogs diagnosed with MUO.

	n = 447
Median age (IQR)	48 months (26‐73)
Median bodyweight (IQR)	7.5 kg (4.5‐11.1)
Sex	
Female	246 (55%)
Male	201 (45%)
Neuter status	
Intact	145 (58.9%)
Neutered	116 (57.7%)
Onset of clinical signs	
Acute	162 (36.2%)
Subacute	120 (26.8)
Chronic	164 (36.7%)
Median duration of clinical signs (IQR)	7 days (3‐14)
Progression of clinical signs	364 (81.4%)
Median neurodisability scale score (IQR)	6 (4‐8)
Median CSF TNCC (IQR)	42 cells/μL (14‐226)
Median CSF protein concentration (IQR)	0.5 g/L (0.3‐1.1)

Abbreviations: CSF, cerebrospinal fluid; IQR, interquartile range; TNCC, total nucleated cell count.

**TABLE 2:** Findings of the neurological examination and presenting neurological signs in 447 dogs diagnosed with MUO.

Neurological deficits	
Proprioceptive deficits	344 (77%)
Ataxia	291 (65.1%)
Obtundation	278 (62.2%)
Paresis^a^	153 (34.2%)
Seizures	144 (32.2%)
Cluster seizures	73/144 (50.7%)
Status epilepticus	35/144 (24.3%)
Cranial nerve deficits	141 (31.5%)
Head tilt	130 (29.1%)
Nystagmus	109 (24.4%)
Tremors	32 (7.2%)
Clinical signs of suspected increased intracranial pressure^b^	55 (12.3%)
Neurolocalization	
Multifocal	194 (43.4%)
Forebrain	131 (29.3%)
Central vestibular	49 (11%)
Brainstem	33 (7.4%)
Cerebellum	17 (3.8%)
Optic nerves/chiasm	17 (3.8%)
No neurological deficits^c^	6 (1.3%)

^a^ Including tetraparesis, hemiparesis, and paraparesis.

^b^ Any combination of the following clinical signs along with mentation changes (obtundation, stupor, or coma): decreased physiological nystagmus, decerebellate, or decerebrate rigidity, pupil size changes.

^c^ Dogs presenting with spinal or unlocalized pain and lethargy.

All dogs received immunosuppressive doses of dexamethasone (0.3‐0.5 mg/kg) at the time of diagnosis (30 dogs did not recover from anesthesia and so did not receive further treatment) alongside cytosine arabinoside in 84.4% (352/417) of dogs (SC injection in 38.8% and IV constant rate infusion in 45.6%). In 22/352 dogs, cytarabine administration was delayed by a few days while waiting for infectious titer results, but it was administered within 1 week of diagnosis.

Median follow‐up time was 11 months (IQR, 1‐24). Eighty‐two percent (366/447) of dogs survived to discharge and 63.5% (284/447) were alive at 6 months. Long‐term follow‐up determined that 55.7% (249/447) of dogs were alive at last follow‐up. Long‐term treatment for the 366 dogs that survived discharge varied substantially among patients in terms of dose and duration of treatment; 14 dogs that had received cytosine arabinoside at diagnosis and were scheduled to have repeated administrations of this medication but died in the 3 weeks after discharge and thus were not included in any of the treatment groups. The treatment groups included 74/352 (21%) dogs treated with corticosteroids alone, 244/352 (69.3%) dogs treated with corticosteroids and repeated SC administrations of cytosine arabinoside, and 34/352 (9.7%) dogs treated with corticosteroids and another PO immunomodulatory drug. Several other immunomodulatory medications were used across the latter 2 groups and included cyclosporine (44), leflunomide (10), azathioprine (7), mycophenolate mofetil (3), procarbazine (2), and lomustine (2). Where this information was clearly identifiable in the clinical records, it was noted that in 36% (103/284) of dogs, despite clinical improvement, resolution of the clinical signs had not occurred by 6 months after diagnosis. A significant difference (*P* = .001) was found between the NDS score on presentation of the dogs that had permanent residual neurological deficits (median, 6; range, 2‐12) compared with those that did not (median, 5; range, 1‐13).

On univariable logistic regression analysis, breed, center, progression of clinical signs, neurolocalization, clinical signs of increased ICP, epileptic seizures, obtundation, paresis, tremors, decreased gag reflex, protocol of cytarabine administration at time of diagnosis, and NDS score showed some evidence of association with survival to 6 months. However, on multivariable analysis, only breed (pugs; *P* = .03), epileptic seizures (*P* < .001), paresis (*P* = .001), and higher NDS score (*P* < .001) at presentation were negatively associated with survival to 6 months (Table 3), with the Hosmer‐Lemeshow statistic indicating acceptable model fit (*P* = .67). The AUC of the NDS score for predicting survival at 6 months after diagnosis of MUO was 0.714 (95% CI: 0.665‐0.764; *P* < .001), suggesting acceptable discrimination. The most suited cut‐off value of the NDS score was 7, with a sensitivity of 61.1% and a specificity of 66.9% for predicting death at 6 months after diagnosis.

**TABLE 3:** Final multivariable logistic regression model for risk factors associated with survival at 6 months after diagnosis in dogs with MUO.

	OR	95% CI	*P* value
Seizures	0.389	0.237‐0.638	<.001*
Paresis	0.422	0.260‐0.685	<.001*
Neurodisability scale score	0.814	0.750‐0.884	<.001*
Breed			.03*
Chihuahua	0.422	0.133‐1.466	.18
Boston terrier	1.653	0.805‐3.393	.17
French bulldog	1.932	0.796‐4.692	.15
Labrador	0.991	0.311‐3.164	.99
Maltese terrier	1.080	0.443‐2.637	.86
Pug	0.255	0.1‐0.653	.004*
West Highland white terrier	1.136	0.452‐2.855	.79
Yorkshire terrier	1.805	0.594‐5.479	.3

Abbreviations: CI, confidence interval; OR, odds ratio.

* Statistically significant.

Relapse occurred in 50.6% (160/316) of dogs that survived to discharge and had detailed follow‐up data (median time to relapse, 7 months; IQR, 2‐15). On univariable Cox regression analysis, age, weight, breed, year of diagnosis, onset and progression of clinical signs, neurolocalization, CSF protein concentration, incomplete resolution of clinical signs during the 6 months after diagnosis, long‐term treatment and NDS score showed some evidence of association with relapse. However, on multivariable Cox regression analysis only incomplete resolution of clinical signs during the 6 months after diagnosis (*P* < .001), higher NDS score (*P* < .001), and >7 days duration of clinical signs before diagnosis (*P* < .001) were associated with relapse (Table 4). Incorporated time‐dependent interaction terms for the final variables were not significant and the Schoenfeld residual plots indicated that the proportional hazards assumption was not violated.

**TABLE 4:** Cox proportional hazards model for risk factors associated with relapse in dogs with MUO.

	HR	95% CI	*P* value
Incomplete resolution of clinical signs	2.441	1.692‐3.523	<.001*
Neurodisability scale score	1.154	1.079‐1.234	<.001*
Duration of clinical signs (≤7 days or ≥7 days)	1.923	1.334‐2.773	<.001*

Abbreviations: CI, confidence interval; HR, hazard ratio.

* Statistically significant.

## 4 DISCUSSION

We identified clinically relevant prognostic variables associated with survival at 6 months after diagnosis, clinical relapse, and long‐term neurological impairment in these dogs with MUO. A total of 63.5% of dogs were alive at 6 months and 50.6% of dogs that survived to discharge subsequently relapsed. The presence of epileptic seizures and paresis at the time of presentation and a higher NDS score were associated with increased risk of death by 6 months after diagnosis. Pugs were also less likely to survive. Incomplete resolution of the clinical signs, higher NDS score, and longer duration of clinical signs were associated with higher risk of relapse. Furthermore, our recently validated NDS was shown to be an important clinical assessment tool because the score was associated with all 3 outcome measures.

The 6‐month survival rate identified in our study is similar to the 69% previously described at 100 days after diagnosis,^6^ and only slightly lower than the 67% to 74% reported 1 week after diagnosis.^4^, ^5^ This finding suggests that patients that survive the initial stage of the disease tend to have a more stable course and respond at least partially to treatment. We also identified that approximately half of the dogs that survive to discharge will relapse, consistent with previous findings with reported relapse rates ranging from 41% to 65%.^4^, ^11^, ^12^, ^13^ Relapses occurred at many different time points and, in some cases, over 12 months after diagnosis. It is therefore likely that many studies with shorter follow‐up times, including ours, fail to identify these patients and that the relapse rate in MUO is in fact higher than has been reported.

The occurrence of epileptic seizures as 1 of the initial clinical signs was associated with decreased survival at 6 months, similar to previous reports.^5^, ^9^, ^10^ Seizures might be associated with a more severe clinical phenotype, but owners also could be more likely to euthanize these patients because of limited tolerance for this clinical sign or the additional treatment entailed and adverse effects. The majority of dogs presenting with seizures had cluster seizures (50.7%) and a substantial proportion (24.3%) had at least 1 episode of status epilepticus. An association between the frequency of cluster seizures and euthanasia has been previously identified in patients with idiopathic epilepsy,^14^ and epilepsy has been shown to affect the quality of life of the dogs and their owners.^15^, ^16^ The importance of adequate anticonvulsive treatment has been emphasized in pugs diagnosed with necrotizing meningoencephalomyelitis (NME) because anticonvulsive drugs were the only treatment associated with longer survival in 1 study.^17^ In view of our results and the previous literature, it is possible that, in MUO patients presenting with seizures, aggressive anticonvulsant treatment to decrease seizure frequency (and therefore improve owner perception of quality of life) ultimately could improve survival.

Obtundation also has been previously reported to be associated with shorter survival times,^5^, ^6^, ^10^ but this association was not found in our population and may reflect the subjectivity involved in evaluation of this clinical sign. We nonetheless identified paresis as another clinical sign associated with decreased survival that has not been reported previously. The presence of motor symptoms at onset of disease has been associated with unfavorable outcome in patients with multiple sclerosis (MS)^18^, ^19^, ^20^, ^21^, ^22^ and several similarities between MS and MUO have been suggested recently.^23^, ^24^, ^25^ It has been suggested that motor symptoms reflect either more extensive or more persistent functional effects or lesions.^26^ Interestingly, no association was found between specific neurolocalizations (such as brainstem) and survival. It is likely that increased risk of death is related to the location of the lesions within the central nervous system, but this association was not identified in our population and may be difficult to establish when the majority of patients present with multifocal signs.

We identified that pug dogs were less likely to survive to 6 months after diagnosis compared with other breeds. This breed is commonly affected by NME,^17^, ^24^, ^27^, ^28^, ^29^ a subtype of MUO that causes extensive necrosis, which could at least partially explain the increase in mortality. Nonetheless, other breeds affected with necrotizing forms of MUO such as the Chihuahua, Maltese, and Yorkshire terrier also were included in the analysis and did not show an increased risk of mortality. Previous studies have reported short survival times for pugs diagnosed with NME but many of these studies included only necropsy‐confirmed cases, which likely biased the results toward more severely affected patients.^17^, ^28^ Serum glial fibrillary acidic protein (GFAP) concentrations were found to be significantly higher in pug dogs with NME compared with healthy controls and other breeds affected with NME, suggesting a breed‐specific fragility of astrocytes.^30^, ^31^ In humans with MS, higher serum concentrations of GFAP were associated with disease severity and higher MRI lesion count,^32^, ^33^ suggesting an important role of astrogliosis in advanced stages of disease.

The NDS has been recently proposed as a reliable clinical scale for initial assessment of patients with MUO.^11^ Our study found an association between the NDS score and increased risk of death within the 6 months after diagnosis, relapse, and incomplete resolution of clinical signs within that time. In human patients, higher baseline score in the expanded disability status scale (EDSS), the most commonly used clinical outcome measure for patients with MS, is predictive for worse outcomes.^20^, ^21^, ^34^, ^35^, ^36^ Identification of a higher EDSS score at baseline increases the level of concern for disease progression in patients with MS and more frequent assessments are recommended in such patients.^37^ Similarly, identification of a higher NDS score at time of diagnosis, especially when >7, should raise concerns over the risk of early death and relapse, and therefore more frequent examinations and repeat MRI when possible should be considered in these dogs. Doing so would allow for more rapid changes to the treatment protocol if necessary, supporting more individual‐based medicine according to the patient's risk profile. The NDS score was found to have good reliability when used prospectively but only moderate reliability when used retrospectively.^11^ Unfortunately, the NDS was not available until recently and, like many clinical studies in veterinary medicine, we relied on retrospective data. Therefore, our initial findings should be interpreted with caution until prospective studies become available. Additional studies should evaluate the NDS for monitoring response to treatment and as an outcome measure. This approach is particularly important in veterinary medicine where the use of survival as an outcome measure is impaired because dogs often are euthanized and owners may elect for euthanasia based on a variety of complex factors such as financial considerations, difficulties managing chronic disease and views on what constitutes acceptable quality of life.

Immunosuppression by use of corticosteroids remains the cornerstone of MUO treatment.^23^ Several other medications have been used, generally with corticosteroids, including cytosine arabinoside, cyclosporine, lomustine, azathioprine, procarbazine, leflunomide, and mycophenolate mofetil.^1^ Unfortunately, large prospective, blinded randomized clinical trials comparing the different treatments directly to each other are not available, which results in a large number of different treatment protocols used. In practice, the treatment protocol often is guided by owner and clinician preference. This situation was reflected in our patient population, where many different combinations of medications and dose regimens were used, which makes interpretation of the results complicated. Currently, no gold standard treatment exists but differences in the treatment protocols (eg, use of corticosteroids alone in some patients) may have influenced the overall findings of our study. Nonetheless, our data reflects common clinical practice and the results therefore should be useful in a clinical setting. Within our study population, no association was found between treatment received at time of diagnosis or long‐term treatment and survival at 6 months after diagnosis on multivariable analysis.

Incomplete resolution of clinical signs at 6 months after diagnosis was associated both with relapse and with the NDS score. It seems plausible that dogs with persistent neurological signs after treatment could have more severe or extensive lesions, which would be reflected in the higher NDS scores. In humans with MS, incomplete recovery also has been shown to be associated with poor prognosis.^19^, ^20^, ^21^, ^38^ Additionally, dogs that presented within 7 days of the onset of clinical signs were less likely to relapse. A previous study had identified that dogs presented within the initial 7 days of clinical signs had longer survival times, but relapse was not evaluated in that population.^8^ It is likely that early treatment may influence outcome as in human patients with MS, starting treatment soon after initial symptom onset minimizes long‐term disability.^39^, ^40^

Our study had some limitations. As previously discussed, the most important limitation was the lack of standardized treatment, which likely had an effect on individual survival times and likelihood of relapse. The NDS score was calculated based on retrospective data available from the patient records and not collected prospectively.^11^ The lack of histopathologic examination in most cases makes misdiagnosis a possibility. The diagnosis of relapse was, for the majority of cases, based on the recurrence of clinical signs and response to changes in the treatment protocol, with only a minority of cases having repeat diagnostic investigations. It is therefore possible that in some cases the clinical signs could have been caused by a second condition. Cases were included over several years and treatment and standards of care may have improved over time, thus influencing outcome. Nonetheless, despite the long study period, there is still no clear evidence of superiority of any specific treatment regimen with multicenter randomized controlled trials required to determine the superiority of a specific treatment.^23^

We showed that pugs, the presence of seizures and paresis at the time of presentation, and a higher NDS score were associated with an increased risk of death at 6 months after diagnosis in dogs with MUO. A higher NDS score, incomplete resolution of the clinical signs, and longer duration of clinical signs before presentation were associated with higher risk of relapse. Awareness of risk factors associated with survival and relapse in MUO can help guide treatment and decision‐making in the management of this debilitating disease. Dogs with recognized risk factors for early death or relapse may benefit from closer monitoring (more frequent re‐examinations by veterinary neurologists) or different treatment protocols. Additional studies are required to determine if this approach would improve the outcome in these high‐risk cases.

## CONFLICT OF INTEREST DECLARATION

Authors declare no conflict of interest.

## OFF‐LABEL ANTIMICROBIAL DECLARATION

Authors declare no off‐label use of antimicrobials.

## INSTITUTIONAL ANIMAL CARE AND USE COMMITTEE (IACUC) OR OTHER APPROVAL DECLARATION

Approved by the IACUC of the University of Liverpool (VREC840).

## HUMAN ETHICS APPROVAL DECLARATION

Authors declare human ethics approval was not needed for this study.
